# Job preferences of master of public health students in China: a discrete choice experiment

**DOI:** 10.1186/s12909-023-04993-9

**Published:** 2024-01-04

**Authors:** Nansheng Luo, Ru Bai, Yu Sun, Xueying Li, Libing Liu, Xin Xu, Li Liu

**Affiliations:** 1 Department of Social Medicine, School of Health Management, China Medical University, No. 77 Puhe Road, Shenyang North New Area, 110122 Shenyang, Liaoning China; 2 Department of Clinical Epidemiology, Shengjing Hospital of China Medical University, No. 36 Sanhao Street, Heping District, 110004 Shenyang, Liaoning China

**Keywords:** Job preference, MPH, Discrete choice experiment, Public health human resources, China

## Abstract

**Background:**

The shortage of public health personnel and the uneven distribution between urban and rural areas are thorny issues in China. Master of public health (MPH) is an integral part of public health human resources in the future, and it is of far-reaching significance to discuss their work preferences. The present study wants to investigate the job preference of MPH, understand the relative importance of different job attributes, and then put forward targeted incentive measures.

**Methods:**

Discrete choice experiment (DCE) was used to evaluate the job preference of MPHs in two medical colleges in Liaoning Province. Attributes include employment location, *bianzhi*, working environment, career development prospects, work value and monthly income. Thirty-six choice sets were developed using a fractional factorial design. Mixed logit models were used to analysis the DCE data.

**Results:**

The final sample comprised 327 MPHs. All the attributes and levels included in the study are statistically significant. Monthly income is the most important factor for MPHs. For non-economic factors, they value career development prospects most, followed by the employment location. Respondents’ preferences are heterogeneous and influenced by individual characteristics. Subgroup analysis showed that respondents from different family backgrounds have different job preferences. Policy simulation suggested that respondents were most sensitive to a salary increase, and the combination of several non-economic factors can also achieve the same effect.

**Conclusions:**

Economic factors and non-economic factors significantly affect the job preference of MPHs. To alleviate the shortage and uneven distribution of public health personnel, more effective policy intervention should comprehensively consider the incentive measures of the work itself and pay attention to the individual characteristics and family backgrounds of the target object.

**Supplementary Information:**

The online version contains supplementary material available at 10.1186/s12909-023-04993-9.

## Background

Since the beginning of this century, the challenges in the public health field have become more severe, and the forms have significantly changed [1]. The outbreak of novel coronavirus at the end of 2019 has spread in more than 200 countries worldwide, causing severe damage to human health, and even causing the whole society to shut down temporarily. Facing public health emergencies, the public health workforce with rich theoretical knowledge and practical abilities plays a crucial role in the health system [2]. To better deal with complex and changeable public health events, it is a crucial step to improve public health education [3]. According to the national health service system planning outline (2015–2020), by 2020, China would have 0.83 public health personnel per 1,000 permanent residents. However, in 2020, this figure was only 0.66. The shortage of public health personnel is indisputable in China [4].

The shortage of health workforce harms the construction of the medical and health system [5]. To promote the development of public health, the Ministry of Education of China launched the full-time Master of Public Health (MPH) postgraduate program in 2009 [6]. According to the China Health Statistics Yearbook in 2018, in terms of educational structure, 54% of the staff in the centres for disease control and prevention at all levels in China have only a junior college degree, 37% had a bachelor’s degree and only 7% had a master’s degree. However, among the public health students worldwide, 37% were undergraduates, 49% were masters, and 14% were doctoral students [7]. Regarding the proportion of highly educated public health personnel, China has not reached the average level in the world. Since the COVID-19 outbreak, China has significantly increased enrollment in MPH.

The uneven distribution of health workers in urban and rural areas is also a concern directly related to people’s access to basic health services [8]. In many countries, there is an uneven distribution of health workers in urban and rural areas or remote areas, and coordinating the balanced health workforce distribution has become a key policy issue [9, 10]. In China, for example, the number of health workers per unit population in urban areas is much higher than in rural areas, and the gap is still widening [11]. The Dakar region, which accounts for only 23% of Senegal’s total population, has more than 60% of the country’s medical staff [12]. About half of the world’s population lives in rural and remote areas, but only a quarter of doctors and less than a third of nurses serve this half [13]. In addition, the WHO report predicts that, over the next few decades, about 40% health professionals worldwide will leave their positions due to little incentives and low wages [14]. Therefore, it is increasingly urgent to formulate reasonable policies and measures to improve the attractiveness of job prospects in rural areas and promote the balanced allocation of urban and rural health human resources.

The public health system plays a vital role in developing China’s health sector and protecting people’s health [15]. Public health is neither solely a hard nor social science and graduates majoring in public health have relatively flexible employment [16]. MPHs have the ability to adjust the focus of work in time according to different environments and can effectively cooperate with other members of the team to realize the complementarity of different majors and improve work efficiency [7]. The job preference of students majoring in health will affect the geographical distribution of health workers in the future [17]. To better allocate public health personnel, it would be a wise choice to investigate the job preference of MPH.

Discrete choice experiment (DCE) can quantitatively measure individual choice preferences [18] by creating simulated choice scenarios [19]. The DCE provides a weighed relevance to the attributes to distinguish highly valued ones [17]. Therefore, quantitative information on the relative strength of selected attributes can be determined, and trade-offs between these attributes and the probability of individuals taking up these jobs can be ascertained [20, 21]. The strength of DCE lies in its ability to assess various scenarios that may not be observed in real-world situations, which makes DCE more widely used in the field of health. For example, scholars in China have studied the employment preferences of pharmaceutical students [22]. In addition, the research on the work preference of community health workers [23] and medical staff [24] has already existed. However, up to now, there is no research on MPHs in China or the world.

In this study, MPHs from two medical universities in Northeast China were taken as the research subjects. The attributes and relative importance of their work preferences were analysed through DCEs. We believe that our findings can provide a basis for public health policymakers to address the unbalanced distribution of human resources in public health and the increasingly severe brain drain phenomenon and improve the construction of China’s health policy systems and mechanisms.

## Methods

### Study context

This study was conducted in Liaoning, a province located in northeast China. Regarding the absolute number of health workers, Liaoning has the most significant number of health workers among the three northern provinces (Liaoning, Jilin, and Heilongjiang) in China. China Health Statistics Yearbook (2020) shows that the number of public health physicians per 10,000 population in Liaoning Province is 0.82, slightly lower than the national average of 0.83.

Considering the number and representativeness of the respondents, we chose the two largest local medical colleges—China Medical University and Dalian Medical University. MPHs, who have yet to graduate, are our target research subjects. Convenience sampling is adopted in this study, and an anonymous web-based survey was conducted through the *Wenjuanxing* platform (one of the most commonly used online questionnaire survey platforms in China) from 8 December 2021 to 2 January 2022.

Previous studies have shown that a small sample size is a significant advantage of DCE. In most cases, a sample size greater than 100 can provide sufficient support for the subsequent analysis of preference data [25]. According to the rule of thumb put forward by Johnson and Orme [26, 27], the sample size of DCE depends on three factors: the maximum number of levels in any attribute (*c*); the number of DCE questions in each questionnaire (*t*); the number of options contained in each DCE question (*a*). The calculation formula is as follows:

$$ N>\frac{500\times c}{t\times a}$$

According to the rule of thumb, the minimum number of respondents required for this study was 63.

### Study design

DCE can design a hypothetical working situation with two options for respondents to choose and determine the relative importance of attributes according to respondents’ choices [28, 29]. The design and analysis of the experiment are in accordance with the user guide of DCE published by the WHO [20] and the instruction manual written by Lancsar [30].

### Identification of attributes and levels

DCE is the most powerful tool to measure individual choice behavior [31]. In a DCE, there are several choice sets, and each choice set is described by specific attributes and levels [32]. The development of attributes and levels is the first and most critical step in DCE experimental design [33]. Through literature review and consideration of the characteristics of public health specialty, we preliminarily determined eight attributes [22, 34–38], including employment location, *bianzhi*, working pressure, working environment, career development prospect, monthly income, training opportunity, and professional compliance. Then, we consulted experts in related majors, and they suggested deleting the attribute of training opportunities because the career development prospect includes training opportunities to some extent. Then, two focus groups were conducted among MPH from China Medical University. They needed to discuss the attributes they would pay attention to when choosing a job. In addition, they also needed to discuss the seven attributes and levels that had been initially determined until they finally reached a consensus. According to their feedback, the attributes of working pressure and professional compliance were deleted, and the attribute of work value was added. Prior to the data collection phase, this study was pre-tested in the MPH of China Medical University, and some minor modifications were made. Finally, six attributes were included in the study, as shown in Table 1. A monetary attribute (monthly income) is always included in DCE to estimate the respondents’ willingness to pay for various attributes [29].

**Table 1:** Attributes and levels used in discrete choice experiment

Attributes	Definition	Levels
Employment location	Employment location refers to medical and health service institutions or other work units in various cities, counties or towns	County
		City
*Bianzhi*	Jobs with *bianzhi* are government-guaranteed positions with lifetime employment and the employees cannot be dismissed by their employers. *Bianzhi* positions are highly valued by the Chinese people because of additional benefits of the position and the sense of belonging it fosters	No
		Yes
Working environment	Work environment mainly refers to social environment such as organisational atmosphere and employee relations	Relatively poor
		Ordinary
		Relatively good
Career development prospects	Career prospects generally refer to the activities such as developmental training and education of knowledge, ability and technology carried out by the organisation, so that individuals can get promotion, respect and achievement to the greatest extent possible	Relatively poor
		Ordinary
		Relatively good
Work value	The value of work mainly includes the public value that individuals create for the society through their work and the personal sense of accomplishment (for example, the work is recognised by the society or the public and promotes the development of the industry)	Low value
		Ordinary
		High value
Monthly income	Monthly income includes wages, bonuses and various welfare subsidies, that is, actual income	RMB 3500
		RMB 5000
		RMB 6500

RMB¥1 = US$0.155, in 2021

### Experimental design

It is helpful to realize unbiased statistical response efficiency by following the standard approaches of DCE design [39]. This study contains six attributes, two of which have a level of 2, and the other four have a level of 3. A full factorial design will produce 324 (2^2^ × 3^4^) potential combinations and 52326 ((324 × 323)÷2) potential choice tasks. The number of potential scenarios presented to the respondents was reduced by a fractional factorial experiment design, and the ‘dcreate’ module of STATA 15.0 was used to optimize the D-efficiency, minimize the overlap among attributes levels, and maximize level balance and orthogonality. The design finally contains 36 choice sets. Studies have shown that the average number of choice sets implemented by DCE for health workers in the past was controlled at about 12 [40]. For reducing the burden of the respondents, the 36 sets were further divided into three blocks, each containing 12 choice sets. An opt-out was included in the second-stage question after each DCE task to allow for unconditional choices [41]. We used an unlabeled DCE for improving the reliability of job preference estimation [42]. Table 2 presents an example of a complete DCE choice set. Respondents need to choose the one they prefer from two hypothetical jobs, and then make a choice whether to choose the job in real life [41]. All three blocks have a duplicate selection set to check the internal consistency, and all respondents were randomized to receive one of the 3 blocks.

**Table 2:** An example of a choice pair

Attributes	Job 1	Job 2
Employment location	City	County
*Bianzhi*	No	Yes
Working environment	Relatively poor	Ordinary
Career development prospects	Ordinary	Relatively poor
Work value	Low value	Ordinary
Monthly income	RMB 3500	RMB 5000
Which job do you prefer?	□	□
Would you choose this job in real life?	□ Yes	□ No

### Data collection

In order to ensure the quality of the research, before the formal investigation, we conducted a small-scale preliminary investigation (*n* = 58) on MPH of China Medical University. In the pre-survey, we tested the questionnaire’s comprehensibility, acceptability and validity. Then, we adjusted the language of the questionnaire and the layout of the questions according to the pre-survey results.

The formal questionnaire consists of three parts: preface (purpose of investigation), demographic information survey and job preference survey. Before the investigation, participants were well-informed about the purpose and protocol of the study, and informed consent was obtained from them. This study was approved by the Ethics Committee of China Medical University.

### Statistical analysis

We use Stata (version 15.0) for data analysis. *χ*^2^ test was adopted to compare category variables in descriptive statistical analysis. DCE data were analysed through mixed logit model, as it has smaller value of the Akaike Information Criterion (AIC) and Bayesian Information Criterion (BIC) than conditional logit model. The smaller the value of the AIC or BIC, the more accurate and reasonable the corresponding regression model in the context of research [43, 44]. All attributes were specified as having a random component. All characteristics were measured as a categorical variable with the first level taken as the reference level, except that the monthly income characteristic was analyzed as a continuous variable. Furthermore, DCEs are based on the random utility theory (RUT) [45]. It assumes that respondent *n* will choose alternative *j* in choice scenario *c* if that alternative provides the most satisfaction out of all other alternatives [46]. It can be specified as:

*U*_*njc*_ = (*β*_*0*_ + *η*_*0n*_) ASC_*njc*_.

+ (*β*_*1*_ + *η*_*1n*_) Employment location _City_.

+ (*β*_*2*_ + *η*_*2n*_) *Bianzhi*_Yes_.

+ (*β*_*3*_ + *η*_*3n*_) Working environment _Ordinary_.

+ (*β*_*4*_ + *η*_*4n*_) Working environment _Relatively good_.

+ (*β*_*5*_ + *η*_*5n*_) Career development prospects _Ordinary_.

+ (*β*_*6*_ + *η*_*6n*_) Career development prospects _Relatively good_.

+ (*β*_*7*_ + *η*_*7n*_) Work value _Ordinary_.

+ (*β*_*8*_ + *η*_*8n*_) Work value _High value_.

+ (*β*_*9*_ + *η*_*9n*_) Monthly income + *ε*_*njc*_.

Where *U*_*njc*_ is the utility of individual *n* from choosing alternative *j* in choice scenario *c*; *β* is a vector of coefficients reflecting the desirability of the attributes; *η* reflects the degree of heterogeneity among respondents; ASC_*njc*_ is the alternative-specific constant; and *ε*_*njc*_ signifies the unobservable random component. An advantage of the mixed logit model relaxes the irrelevant alternatives (IIA) assumption by allowing coefficients to vary between individuals, thus it can consider the potential heterogeneity of respondents’ preferences [47]. Furthermore, in this model the desirability of attributes constitutes a vector of average preferences of the population for each attribute (*β*) and the individual’s specific preference components (*η*) [48]. Both the mean and standard deviation (SD) of *β* will be estimated, from which preference heterogeneity can be assessed [49].

When there are economic attributes (such as monthly income) in each set research attribute, by calculating the ratio of regression coefficients of economic attributes and non-economic attributes we can get the evaluation of the monetary value of each non-economic attribute (i.e. willingness to pay) of the respondents. The willingness to pay (WTP) can measure the income that the respondents are willing to give up in order to get a certain attribute or the compensation income expected by accepting a certain attribute [50]. A simulation study was conducted to predict that the uptake rates of MPH for rural versus city jobs change as the levels of job attributes are changed.

## Result

A total of 401 MPHs filled out the questionnaires through the *wenjuanxing* platform. Thirty-four (8.5%) respondents were rejected because of their short answer time (an answer time of less than 50% of the median answer time will be defined as answering too fast) [51]. There are also 40 (10.0%) respondents who were excluded because they failed the internal consistency test. In the end, the total number of valid questionnaires in this study was 327.

### Characteristics of the respondents

Table 3 presents the basic information of the respondents included in the analysis. The average age of the MPH (*n* = 327) was 24.2 ± 1.8 years. Female (77.68%) accounts for the overwhelming majority of participants, which was consistent with a study on the employment intention of Chinese students majoring in public health [52]. There are 191 (58.41%) people from non-only-child families, which is slightly higher than those from only-child families. Most were grade 1 master respondents (48.62%) and from rural areas (43.73%). About 78.59% of the students plan to work in public health and preventive medicine after graduation, and about half are neutral about their future employment forms. See Table 3 for more details.

**Table 3:** Characteristics of the respondents (*n* = 327)

Characteristics	*n*	%
Age (year), Mean ± SD	24.17 ± 1.75	
Gender		
Male	73	22.32%
Female	254	77.68%
Single child		
Yes	136	41.59%
No	191	58.41%
Degree type		
Academic degree	55	16.82%
Professional degree	272	83.18%
Grade		
Grade 1	159	48.62%
Grade 2	125	38.23%
Grade 3	43	13.15%
Monthly consumption (RMB)		
< 800	12	3.67%
800–1200	94	28.75%
1200–1600	107	32.72%
1600–2000	69	21.10%
> 2000	45	13.76%
Place of origin		
Rural	143	43.73%
Country	83	25.38%
City	101	30.89%
Annual family income (RMB)		
< 50,000	88	26.91%
50,000–100,000	131	40.06%
100,000–150,000	69	21.10%
150,000–200,000	28	8.56%
> 200,000	11	3.36%
Post-graduation planning		
Public health and preventive medicine related work	257	78.59%
Continue to study	59	18.04%
Start a business	2	0.61%
Other	9	2.75%
Attitude towards employment forms		
Very pessimistic	21	6.42%
Pessimistic	90	27.52%
Neutral	161	49.24%
Optimistic	7	2.14%
Very optimistic	48	14.68%

SD, standard deviation. RMB¥1 = US$0.155, in 2021

### Mixed logit estimates for MPH’s job preference

**Fig. 1:**
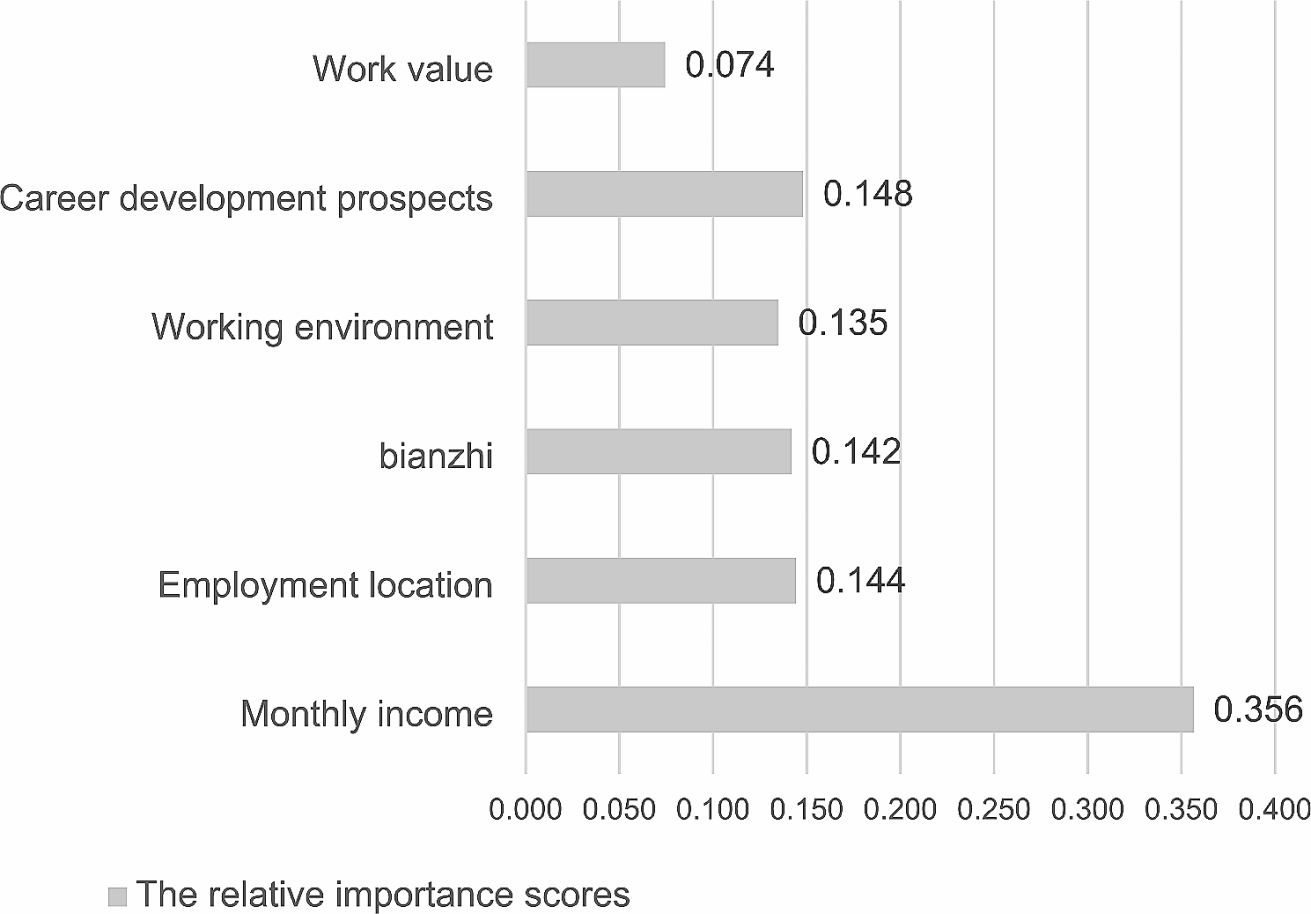
The relative importance scores of each attribute. Higher scores indicate that the attribute is more important to the respondents $({\rm{the}}\,{\rm{relative}}\,{\rm{importance}}\,{\rm{scores}}\,{\rm{of}}\,{\rm{attribute}}\,i = \frac{{{\rm{Maximum}}\,{\rm{utility}}\,{\rm{of}}\,{\rm{attribute}}\,i}}{{{\rm{Total}}\,{\rm{utility}}}})$

The results of the regression analysis of the mixed logit model are shown in Table 4. Among the six attributes included in the study, at least one level of each attribute is statistically significant, which means that all these attributes are crucial to the job preference of MPH. The sign of each regression coefficient implies that the respondents have obtained a higher utility level from a higher attribute level and made a rational choice [53]. In choosing a job, MPH considers not only economic factors but also non-economic factors. They pay special attention to their career development prospects (*β* = 1.046, *p* < 0.001) and strongly prefer jobs with relatively good career development prospects rather than relatively poor ones. The employment location (*β* = 1.019, *p* < 0.001) is also their key attribute, and they tend to seek a job in the city rather than in the country. The alternative specific constant (ASC) for the opt-out is significantly negative, which indicates that the respondents are more likely to express their preference for choosing a job, regardless of the attributes and level presented [54], that is, they show a positive attitude towards choosing a job. For monthly income, employment location, *bianzhi*, working environment and relatively good career development prospects, the SDs of those random coefficients were statistically significant, which indicated preference heterogeneity was present for those attribute levels. The relative importance scores of each attribute are calculated and presented in Fig. 1. As we can see from the Figure, the monthly income and career development prospects dominate the choice of a job for MPH. Work value is regarded as the least important attribute, with a score of 0.074.

**Table 4:** Mixed logit estimates for MPH’s job preference

Attributes and levels	Mean		SD	
	Coefficient	*p*-value	Coefficient	*p*-value
ASC (out-put)	-6.334	< 0.001	3.181	< 0.001
Monthly income	0.000839	< 0.001	0.000326	< 0.001
Employment location (ref: Country)				
City	1.019	< 0.001	1.293	< 0.001
*Bianzhi* (ref: No)				
Yes	1.002	< 0.001	1.031	< 0.001
Working environment (ref: Relatively poor)				
Ordinary	0.543	< 0.001	-0.381	0.014
Relatively good	0.951	< 0.001	-0.452	0.005
Career development prospects (ref: Relatively poor)				
Ordinary	0.564	< 0.001	-0.288	0.198
Relatively good	1.046	< 0.001	0.698	< 0.001
Work value (ref: Low value)				
Ordinary	0.178	0.038	-0.065	0.692
High value	0.526	< 0.001	-0.285	0.095
Sample size	327			
Number of observations	11,772			
Log likelihood	-2911			
AIC	5862			
BIC	6009			

SD, standard deviation; Likelihood simulated using 1000 Halton draws; AIC, Akaike information criterion; BIC, Bayesian information criterion

### Willingness to pay for job attributes

The results of WTP estimates are documented in Table 5. The most crucial job attributes, ranked in terms of willingness to sacrifice monthly income, are career development prospects, employment location and *bianzhi*. They were willing to pay RMB 1246.3 (US$ 184.6) monthly income for a job with relatively good career development prospects than a job with relatively poor career development prospects. In order to leave the country to work in the city, they are willing to give up RMB 1214.5 (US$ 179.9) per month.

**Table 5:** Estimated willingness to pay (WTP) for job attributes among MPH

Attributes and levels	WTP (RMB)	95% confidence interval	
		Lower level	Upper level
Employment location (ref: Country)			
City	1214.5	951.0	1478.0
*Bianzhi* (ref: No)			
Yes	1194.0	963.7	1424.3
Working environment (ref: Relatively poor)			
Ordinary	647.4	440.1	854.7
Relatively good	1133.9	888.5	1379.2
Career development prospects (ref: Relatively poor)			
Ordinary	672.6	468.7	876.6
Relatively good	1246.3	988.0	1504.6
Work value (ref: Low value)			
Ordinary	212.2	9.2	415.1
High value	626.6	414.9	838.2

WTP, willingness to pay; RMB¥1 = US$0.155, in 2021

### Subgroup analysis

In view of our analysis of the existing literature and the results of focus group interviews, this study carried out a subgroup analysis on the respondents based on whether they were only child, where their families belonged, and the total annual income of their families. We found that there were differences between the job preference and the willingness to pay of related attributes of respondents with different individual characteristics. Respondents from only child families pay the most attention to the employment location when choosing jobs. It is worth noting that respondents whose families belong to cities and counties and whose total annual income is more than RMB 100,000 (US$ 14821.8) all show the same preference. In addition, the most crucial job attribute for respondents whose total annual household income is less than RMB 100,000 is *bianzhi*. They are willing to pay RMB 1169.3 (US$ 173.3) for a job with *bianzhi*. More details are provided in Additional file 1: Supplementary Tables 1–3. Considering the difficulty and efficiency of model fitting, basic demographic variables were not included in our subgroup analysis. This will be further discussed in the follow-up study.

### Changes in employment rate under different policy interventions

**Fig. 2:**
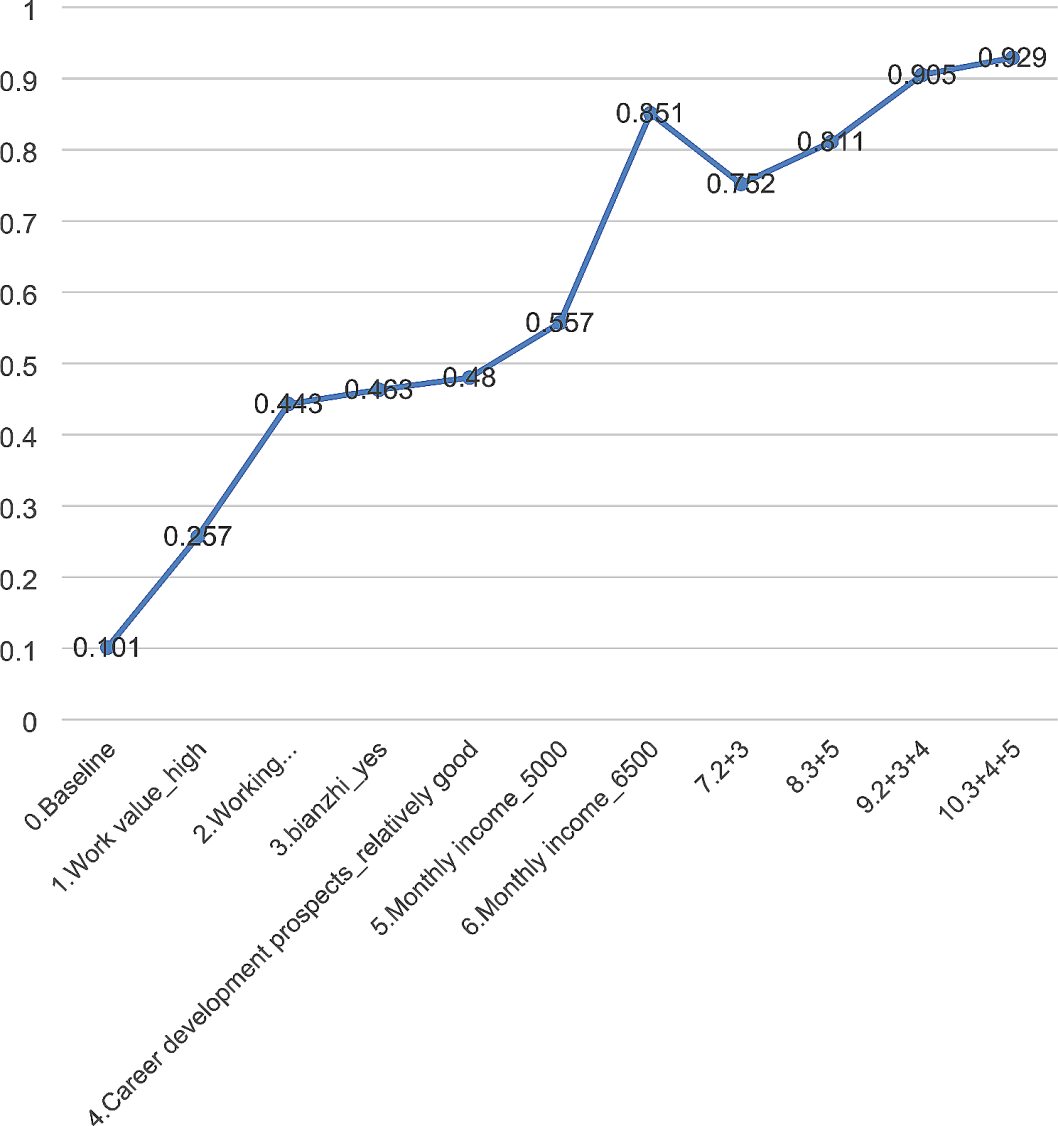
The changes in the probability of taking a job in country. With the improvement of working attributes, the probability of MPH willing to work in remote areas changes. Each serial number represents the corresponding attribute, and the combination of serial numbers represents the combination of different attributes (Baseline: Monthly income_RMB 3500, *Bianzhi*_No, Working environment_Relatively poor, Career development prospects_Relatively poor, Work value_Low value)

Figure 2 shows the changes in the probability of taking a job in country. It can be seen from the figure that under the benchmark condition (Monthly income: RMB 3500, *Bianzhi*: No, Working environment: Relatively poor, Career development prospects: Relatively poor, Work value: Low value), the probability of MPHs choosing to work in counties and towns is only 10.1%. Holding all else the same, if monthly income increased from RMB 3500 to RMB 5000, the probability of choosing a country job would increase to 55.7%. When the three non-economic factors, such as employment location, *bianzhi* and career development prospects, change at the same time (9: 2 + 3 + 4), the probability of MPH choosing to work in counties increases to 90.5%, slightly higher than the probability of working in cities under the benchmark conditions.

## Discussion

The present study was the first DCE study conducted among MPHs to investigate their preferences in job attributes. DCE is helpful as it can check the respondents’ declarative preference and willingness to choose rather than their explicit preference (actual choice) [55]. In this study, we finally determined six attributes closely related to MPH’s job preference. Mixed Logit analysis showed that all the attributes and levels were statistically significant, which is consistent with other studies [18, 24]. Monetary attribute has the most significant influence, consistent with the results of research on job preference conducted in many other places [23, 37, 47]. The employment location is one of the most crucial nonmonetary attributes of MPH. There is a big gap between rural and urban areas in terms of infrastructure and educational opportunities for children, which may be one of the reasons for the shortage of rural health human resources [56]. Similar results have been reported in other human resource DCE studies [22]. Lehmann et al. have also pointed out that students’ home location will have an impact on their future career choice [56]. Therefore, when formulating relevant policies to attract MPH to work in remote areas, the family background of the target object should be considered.

*Bianzhi* is a strong motivator of MPH. Researches on job preference in China generally includes this attribute and usually has a relatively significant influence [57, 58]. Chinese jokingly call *bianzhi* “iron rice bowl”, which means that the work with *bianzhi* has strong stability and will likely accrue better job security [59]. Chen surveyed employment intention in medical higher vocational colleges [60]. The study showed that students in areas with relatively backward economic and cultural development levels have higher requirements for job stability, and our research found a similar result. The most crucial job attribute for students from relatively poorer families is the *bianzhi*, while for students from relatively wealthier families is the employment location. For policymakers, in order to attract MPH to devote themselves to the development of rural health undertakings, it may be more effective to provide preparation for students from relatively low-income families.

As expected, the essential attribute for all the respondents is the economic factor. As we can see from the simulated working situation, by raising the monthly income level by one level (from RMB 3500 to RMB 5000), the respondents’ willingness to work in counties will increase by 45%. At present, China’s health service personnel are not satisfied with the salary, and improving income and welfare will have positive significance for retaining them [61, 62]. The monthly income level of workers is significantly related to the financial strength of the employment location [63]. Considering that the economic level and development level of counties are generally lower than those of cities, it is a more practical method to combine economic incentives with non-economic incentives.

Work value is the least valued attribute of MPH. According to Maslow’s Hierarchy of Needs [64], the desire of the previous level is satisfied, there is a chance to satisfy the desire of the next level. The pursuit of work value is a manifestation of self-realization, so it can be considered that its priority will be lower than other attributes. McAuliffe et al. pointed out in their research on the work preference of health workers providing obstetric care that once work reward can meet their basic needs, and other job attributes will become more important than salary [65]. Therefore, it can be inferred that the respondents have no confidence in the welfare of their future jobs. When making policies to attract MPH to relatively remote and backward areas, it is necessary to meet their basic living needs.

When formulating targeted recruitment policies, it is necessary to understand the individual characteristics of the respondents fully. Research has shown that gender has a significant impact on job selection [22, 66]. According to our research, respondents from only-child families prefer to work in cities and relatively good working environments. Two explanations are available. Firstly, the one-child families are more concentrated in urban areas [67]. Because of the influence of students’ geographical origin on their career choice [56], students from urban areas are more inclined to work in cities [34]. Secondly, the economic conditions of the only-child families are relatively better [68], and their children will be more inclined to choose places with better supporting facilities and more convenient life. Thus, more attention can be paid to the applicant’s family situation when recruiting MPH in underdeveloped counties and towns.

The research has the following three limitations. Firstly, the coverage of the sample is not wide enough and the representativeness is slightly insufficient. Secondly, this study focused on job seekers. For formulating more accurate and effective policies and measures, preference of the employer should also be considered. Thirdly, we did not explore differences in the results between the two institutions due to the limited sample size. However, the learning environment of the respondents often has an important impact on their career planning, and the differences should be explored in future studies.

## Conclusions

The present study found that monthly income and employment location are two crucial job attributes in MPH’s career selection process. MPH with different individual characteristics has different preferences for job attributes. Combining economic and non-economic factors could be a more effective and feasible measure. Our results suggest a variety of possibilities to improve MPHs’ deployment in rural settings. The findings of this study will help policymakers to design a more effective recruitment plan for MPH in China, alleviate the shortage and uneven distribution of public health personnel, and thus construct a more reasonable public health system.

### Electronic supplementary material

Below is the link to the electronic supplementary material.

**Supplementary Material 1: Supplementary Table 1.** Results of mixed logit models and WTP (Single child). **Supplementary Table 2.** Result of mixed logit models and WTP (Place of origin). **Supplementary Table 3.** Result of mixed logit models and WTP (Annual family income (RMB))

## Data Availability

The datasets used or analysed during the current study are available from the corresponding author on reasonable request.

## Abbreviations

AIC: Akaike information criterion
BIC: Bayesian information criterion
DCE: Discrete choice experiment
MIXL: Mixed logit model
MPH: Master of Public Health
RUT: Random utility theory
