# Supplementary material for: Job preferences of master of public health students in China: a discrete choice experiment
Source: BMC Med Educ. 2024 Jan 4;24:24. doi: 10.1186/s12909-023-04993-9 (PMC10768294; doi:10.1186/s12909-023-04993-9)
Supplement: Supplementary file 1 — Supplementary Material 1: Supplementary Table 1. Results of mixed logit models and WTP (Single child). Supplementary Table 2. Result of mixed logit models and WTP (Place of origin). Supplementary Table 3. Result of mixed logit models and WTP (Annual family income (RMB)) [file 12909_2023_4993_MOESM1_ESM.docx]

**Supplementary Table 1.** Results of mixed logit models and WTP (Single child)

| **Attributes and levels** | **Single child-Yes** | | | **Single child-No** | | |
| --- | --- | --- | --- | --- | --- | --- |
|  | **Coefficient** | ***p*-value** | **WTP(RMB)** | **Coefficient** | ***p*-value** | **WTP(RMB)** |
| ASC (out-put) | -8.183 | <0.001 |  | -5.338 | <0.001 |  |
| Monthly income | 0.000913 | <0.001 |  | 0.000807 | <0.001 |  |
| Employment location (ref: Country) | | | | | | |
| City | 1.490 | <0.001 | 1631.5 | 0.712 | <0.001 | 882.5 |
| *Bianzhi* (ref: No) |  |  |  |  |  |  |
| Yes | 1.065 | <0.001 | 1166.1 | 0.961 | <0.001 | 1190.9 |
| Working environment (ref: Relatively poor) | | | | | | |
| Ordinary | 0.711 | <0.001 | 778.2 | 0.442 | <0.001 | 548.4 |
| Relatively good | 1.272 | <0.001 | 1393.2 | 0.731 | <0.001 | 906.0 |
| Career development prospects (ref: Relatively poor) | | | | | | |
| Ordinary | 0.699 | <0.001 | 765.3 | 0.474 | <0.001 | 587.0 |
| Relatively good | 1.148 | <0.001 | 1256.6 | 0.981 | <0.001 | 1215.8 |
| Work value (ref: Low value) | | | | | | |
| Ordinary | 0.272 | 0.063 | 298.3 | 0.119 | 0.265 | 147.6 |
| High value | 0.605 | <0.001 | 662.0 | 0.465 | <0.001 | 577.0 |

WTP, willingness to pay; RMB￥1 = US$0.155, in 2021; ref, reference

**Supplementary Table 2.** Result of mixed logit models and WTP (Place of origin)

| **Attributes and levels** | **Place of origin-Country and city** | | | **Place of origin-Rural** | | | |
| --- | --- | --- | --- | --- | --- | --- | --- |
|  | **Coefficient** | ***p*-value** | **WTP(RMB)** | **Coefficient** | ***p*-value** | **WTP(RMB)** | |
| ASC (out-put) | -8.058 | <0.001 |  | -4.742 | <0.001 |  |  |
| Monthly income | 0.000912 | <0.001 |  | 0.000784 | <0.001 |  |  |
| Employment location (ref: Country) | | | | | | |  |
| City | 1.631 | <0.001 | 1789.2 | 0.490 | <0.001 | 625.2 |  |
| *Bianzhi* (ref: No) |  |  |  |  |  |  |  |
| Yes | 1.317 | <0.001 | 1444.8 | 0.738 | <0.001 | 941.0 |  |
| Working environment (ref: Relatively poor) | | | | | | |  |
| Ordinary | 0.680 | <0.001 | 745.3 | 0.413 | 0.001 | 526.5 |  |
| Relatively good | 1.195 | <0.001 | 1310.4 | 0.705 | <0.001 | 898.8 |  |
| Career development prospects (ref: Relatively poor) | | | | | | |  |
| Ordinary | 0.535 | <0.001 | 586.4 | 0.563 | <0.001 | 718.4 |  |
| Relatively good | 1.277 | <0.001 | 1401.0 | 0.792 | <0.001 | 1009.3 |  |
| Work value (ref: Low value) | | | | | | |  |
| Ordinary | 0.238 | 0.057 | 261.0 | 0.100 | 0.401 | 127.7 |  |
| High value | 0.596 | <0.001 | 654.2 | 0.410 | 0.001 | 522.8 |  |

WTP, willingness to pay; RMB￥1 = US$0.155, in 2021; ref, reference

**Supplementary Table 3.** Result of mixed logit models and WTP (Annual family income (RMB))

| **Attributes and levels** | **Annual family incomes**  **(RMB): < 100000** | | | | **Annual family incomes**  **(RMB): > 100000** | | | | |
| --- | --- | --- | --- | --- | --- | --- | --- | --- | --- |
|  | **Coefficient** | | ***p*-value** | **WTP(RMB)** | **Coefficient** | | ***p*-value** | **WTP(RMB)** | |
| ASC (out-put) | -5.456 | <0.001 | |  | -8.452 | <0.001 | |  |  |
| Monthly income | 0.000838 | <0.001 | |  | 0.000863 | <0.001 | |  |  |
| Employment location (ref: Country) | | | | | | | | |  |
| City | 0.853 | <0.001 | | 1017.2 | 1.440 | <0.001 | | 1668.1 |  |
| *Bianzhi* (ref: No) |  |  | |  |  |  | |  |  |
| Yes | 0.980 | <0.001 | | 1169.3 | 1.081 | <0.001 | | 1252.1 |  |
| Working environment (ref: Relatively poor) | | | | | | | | |  |
| Ordinary | 0.440 | <0.001 | | 525.0 | 0.814 | 0.001 | | 943.8 |  |
| Relatively good | 0.870 | <0.001 | | 1038.2 | 1.162 | <0.001 | | 1345.9 |  |
| Career development prospects (ref: Relatively poor) | | | | | | | | |  |
| Ordinary | 0.495 | <0.001 | | 590.2 | 0.748 | <0.001 | | 866.4 |  |
| Relatively good | 0.956 | <0.001 | | 1141.0 | 1.291 | <0.001 | | 1496.2 |  |
| Work value (ref: Low value) | | | | | | | | |  |
| Ordinary | 0.077 | 0.457 | | 92.4 | 0.423 | 0.007 | | 489.9 |  |
| High value | 0.383 | <0.001 | | 457.3 | 0.827 | <0.001 | | 958.1 |  |

WTP, willingness to pay; RMB￥1 = US$0.155, in 2021; ref, reference
